# Supplementary material for: MetaRibo-Seq measures translation in microbiomes
Source: Nat Commun. 2020 Jun 29;11:3268. doi: 10.1038/s41467-020-17081-z (PMC7324362; doi:10.1038/s41467-020-17081-z)
Supplement: Supplementary file 10 — Supplementary Data 7 [file 41467_2020_17081_MOESM10_ESM.zip › File2/Confidence_High_Taxonomy/221357_out.krona.html]

Javascript must be enabled to view this page.

members
magnitude
magnitudeUnassigned
count
unassigned
taxon
rank

221357\_out

4

4
superkingdom
2

4
1239
phylum

class
186801
1

order
186802
1

1
family
543314

1
genus
86331

species
114527

SRS011306\_contig\_number\_116
1

909932
class
3

order
909929
3

3
family
1843491

genus
970
3

712538
species
1

SRS144300\_contig\_number\_11609


SRS1041121\_contig\_number\_1146
1
69823
species

species
671224

SRS893232\_contig\_number\_13
1
